# Quantitative Analysis of Soil Total Nitrogen Using Hyperspectral Imaging Technology with Extreme Learning Machine

**DOI:** 10.3390/s19204355

**Published:** 2019-10-09

**Authors:** Hongyang Li, Shengyao Jia, Zichun Le

**Affiliations:** 1 College of Computer Science and Technology, Zhejiang University of Technology, Hangzhou 310023, China; freelhy@126.com; 2 College of Mechanical and Electrical Engineering, China Jiliang University, Hangzhou 310018, China; 15a0106134@cjlu.edu.cn; 3 College of Science, Zhejiang University of Technology, Hangzhou 310023, China

**Keywords:** hyperspectral imaging, soil total nitrogen, partial least squares, extreme learning machine, uninformative variable elimination, successive projections algorithm

## Abstract

Soil nutrient detection is important for precise fertilization. A total of 150 soil samples were picked from Lishui City. In this work, the total nitrogen (TN) content in soil samples was detected in the spectral range of 900–1700 nm using a hyperspectral imaging (HSI) system. Characteristic wavelengths were extracted using uninformative variable elimination (UVE) and the successive projections algorithm (SPA), separately. Partial least squares (PLS) and extreme learning machine (ELM) were used to establish the calibration models with full spectra and characteristic wavelengths, respectively. The results indicated that the prediction effect of the nonlinear ELM model was superior to the linear PLS model. In addition, the models using the characteristic wavelengths could also achieve good results, and the UVE–ELM model performed better, having a correlation coefficient of prediction (r_p_), root-mean-square error of prediction (RMSEP), and residual prediction deviation (RPD) of 0.9408, 0.0075, and 2.97, respectively. The UVE–ELM model was then used to estimate the TN content in the soil sample and obtain a distribution map. The research results indicate that HSI can be used for the detection and visualization of the distribution of TN content in soil, providing a basis for future large-scale monitoring of soil nutrient distribution and rational fertilization.

## 1. Introduction

Soil is an important part of agricultural production. Scientific fertilization according to the richness or poorness of nutrients in the soil is the basis for high-quality and high-yield crops [1]. However, fertilization is often done blindly or mechanically in order to obtain a high yield, resulting in the uneven distribution and low utilization of chemical fertilizers. Excessive nitrogen fertilizer use not only reduces the rate of fertilizer utilization and causes economic losses but also causes serious environmental pollution and excessive nutrient content in crops [2].

Traditional methods for detecting soil nutrients are done through laboratory chemical analysis tests [3]. However, these analyses are time consuming, expensive, and complicated to operate. In addition, the acid–base waste liquid produced by the laboratory can cause secondary environmental pollution if improperly handled. Therefore, there is an urgent need for a rapid, on-site, continuous, and nonpolluting detection method for crop production. A method that can quickly and accurately obtain the content and distribution of total nitrogen (TN) in soil, so as to rationally and precisely fertilize according to the abundance of soil nutrients [4], is of great significance for the sustainable development of agriculture and the successful implementation of precision agriculture.

Near-infrared (NIR) spectroscopy has been widely used in the detection of soil nutrient information due to its advantages of fast detection speed, no pollution, low cost, and simple operation [5]. He et al. used NIR spectroscopy combined with partial least squares (PLS) to analyze the contents of nitrogen (N), phosphorus (P), potassium (K), organic matter (OM), and pH in the soil, and the results showed that NIR had the potential to accurately predict N and OM contents as well as pH level in soil [6]. Rossel et al. compared linear regression analysis methods (i.e., multiple linear regression, partial least squares, etc.) with nonlinear regression analysis methods (i.e., support vector machines (SVMs), random forests (RFs), artificial neural networks, etc.) and conducted modeling research on the content of soil organic carbon (OC) and clay [7]. The results of this study indicated that the model established using a nonlinear data mining algorithm could achieve a better prediction effect. The prediction model based on the extreme learning machine (ELM) algorithm has strong analytical ability and robustness for nonlinear problems and has been successfully applied in many fields [8,9]. In soil detection, ELM has also been widely used to estimate soil moisture, soil temperature, and soil organic matter, for which it has achieved high prediction accuracy [10,11,12].

Hyperspectral imaging (HSI) is a widespread, rapid, and nondestructive analytical technology which organically combines traditional spectral analysis with image processing. HSI can simultaneously obtain the continuous spectral information of each pixel in a sample image and the continuous image information of each wavelength in the spectrum [13]. Spectral information can reflect the molecular structure and composition state of a sample, while the image information can reflect its appearance characteristics. Therefore, a hyperspectral image can provide the sample’s spectral and spatial information at the same time. O’Rourke et al. used hyperspectral technology to detect the OM and OC contents of forest surface soils [14]. Jiang et al. used hyperspectral technology to carry out quantitative estimation and comparison of the cadmium (Cd) concentration of standard and naturally Cd-contaminated soil samples [15]. However, these researchers only used the spectral information in hyperspectral data for modeling and prediction, while image information in HSI technology can spatially reflect the distribution of the reference values of samples in order to make corresponding decisions according to the distribution map. This method has been successfully applied in many fields [16,17,18]. In this work, the spectral and image information in hyperspectral data was used to detect soil nutrient content and obtain content distribution maps.

This study explored different methods of using HSI technology to detect and visualize the distribution map of TN content in soil. The specific objectives were (1) to establish an ELM model with a good predictive effect for predicting TN content in soil, (2) to compare the prediction effects of corresponding models under different characteristic wavelength selection methods to determine the optimal model for predicting TN content in soil, and (3) to detect the TN content and visualize its distribution map based on the optimal model.

## 2. Materials and Methods

### 2.1. Soil Samples

In this work, the research area is located in Lishui City (27°25′–28°57′ N, 118°41′–120°26′ E), Zhejiang Province, the People’s Republic of China. The area belongs to the mid-subtropical monsoon climate zone, and the terrain is dominated by mountains and hills. According to the classification and codes for Chinese soil (National Standard of China, GB/T 17296–2009), the representative soil type in this area is paddy soil.

**Figure 1:**
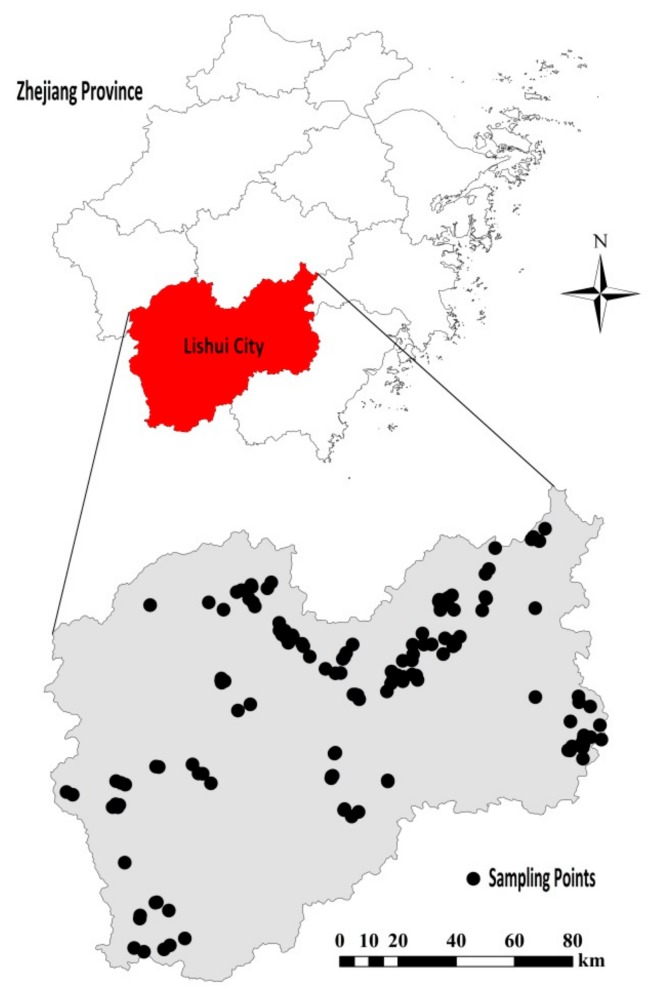
Geographical map of the research area and locations of sampling points.

A total of 150 paddy soil samples were taken from different farmlands of eight counties in Lishui (Figure 1). Since there were many impurities such as weeds and stones in the soil surface layer, soil samples were taken from the surface layer (5–20 cm) in order to reduce the impact of impurities on the measurement results. We adopted a five-point sampling method. A square region (1 × 1 m) was selected at first. Then, an undisturbed earth drill was used for sampling at the four endpoints and the center of the region at a total of five positions. Finally, five soil samples of equal volume were mixed to obtain a soil sample. For the sake of reducing the impact of soil moisture and soil particle size on the measurement results, the soil was first air-dried in a cool and ventilated place to remove plant residue and stones from the soil. Then, the soil was milled and sieved, with a sieve aperture of 2 mm. Finally, the soil samples were divided into two parts. The Institute of Soil Fertilizer (Zhejiang Academy of Agricultural Sciences) carried out a chemical analysis on a small portion (about 50 g) of each soil sample to measure the laboratory reference values of TN content in the soils. The remaining samples were stored in petri dishes for HSI measurement and data analysis. The laboratory reference values of TN content in soil were determined using the Kjeldahl method [19]. The reference value of TN is the percentage of the TN’s weight out of the total weight of the dry soil sample.

From the 150 soil samples, 100 samples were randomly selected as the calibration set, and the remaining 50 samples were used as the prediction set. The reference values of soil TN content are shown in Table 1. The range of TN content in the calibration set contained the range of TN content in the prediction set.

### 2.2. Hyperspectral Imaging System

**Figure 2:**
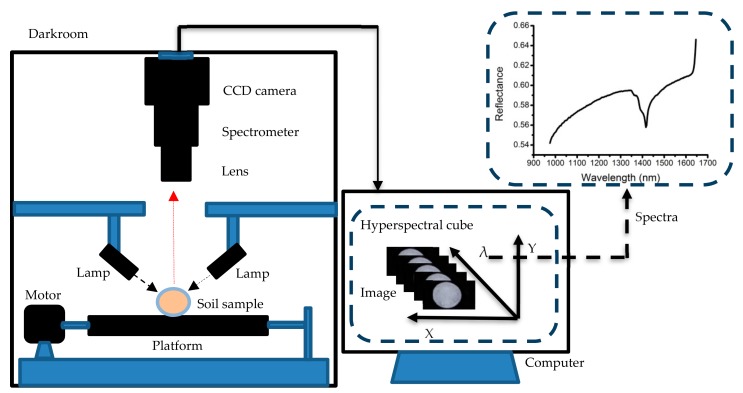
The hyperspectral imaging (HIS) system.

A near-infrared HSI system was used for the spectral measurement of soil samples (Figure 2). From top to bottom, the system was composed of a CCD camera (Xeva 992; Xenics Infrared Solutions, Leuven, Belgium), a spectrometer (ImSpector N17E, Spectral Imaging Ltd., Oulu, Finland) with a spectral range of 900–1700 nm (256 wavelengths), an imaging lens (OLES22, Specim, Spectral Imaging Ltd., Oulu, Finland), a lighting source assembled using two 150 W fiber halogen lamps (Fiber-Lite DC950 Illuminator, Dolan Jenner Industries Inc., Boxborough, MA, USA) with adjustable intensity from 0% to 100%, and a single-phase stepper motor (Isuzu Optics Corp., Taiwan) for driving the displacement platform.

The soil sample was placed in a 60 mm diameter petri dish, then the petri dish was placed on the platform for image acquisition. To reduce the impact of environmental light on the sample while measurements were being taken, the entire system (except the computer) was assembled in a darkroom. In addition, in order to obtain a complete and undistorted hyperspectral image, the translational speed of the platform was set to 24 mm/s, the camera exposure time was set to 3 ms, and the soil sample was placed 30.8 cm below the camera lens.

### 2.3. Image Preprocessing 

In order to reduce the impact of changes in camera dark current and light intensity on the image, the original image (*I_0_*) acquired by the HSI system was corrected using dark and white plate reference images. The white reference image (*I_w_*) was acquired by scanning a white Teflon brick with a reflectance close to 100%, while the dark reference image (*I_d_*) was acquired by shutting down all lamps and covering the camera lens with its own opaque lens cap to get nearly 0% reflectance.

The corrected image *I* was obtained using Equation (1):

(1) $$I=\frac{I_{0}-I_{d}}{I_{w}-I_{d}}$$

For the corrected hyperspectral images of the soil samples in each petri dish, an area with a size of 50 × 50 pixels in the center of each image was selected as the region of interest (ROI) [20]. The average reflection spectrum of all pixels in the ROI was calculated as the mean spectral data of the sample. Due to the influence of noise at both ends of the spectrum, only the spectrum of 975–1645 nm (200 wavelengths) was selected for further analysis and modeling. 

### 2.4. Multivariate Data Analysis 

As the spectral data obtained by the HSI system has complex background noise and severe peak overlap, direct analysis is very difficult. Therefore, it is necessary to use chemometric methods to extract effective information from spectral data [21]. Regression analysis was mainly used to study the functional relationship between variables and establish a regression model for further analysis and prediction [22]. In spectroscopy and image analysis, the reference values of the object are studied by using spectral and image information, and the inner relations are studied to establish a calibration model for predicting unknown samples. In this work, PLS in linear regression analysis and ELM in nonlinear regression analysis were used to build the calibration model.

#### 2.4.1. Modeling Methods 

The PLS algorithm was first proposed by Wold et al. (1985). PLS is a common regression method to solve the problem of data multicommonness by extracting data characteristic information to realize data compression [23]. The PLS algorithm considers both spectral information (*X*) and the corresponding reference values (*Y*) of samples during modeling and transforms the original spectral data into mutually orthogonal and unrelated new variables via linear transformation, thereby eliminating multicollinearity between datasets. The new variables are a linear combination of the original data, called latent variables (LVs). In the calculation of LVs, the variance of LVs should not only be as large as possible but the correlation between LVs and reference values should also be maximized. Based on the calculation, the first few LVs carry most of the primary information, which are used to replace the original spectral data and establish the calibration model [24]. In this work, the optimal number of LVs used in the PLS model was decided by cross validation with Unscrambler 9.7 software (CAMO Inc., Oslo, Norway).

The ELM is a new single-hidden-layer, feedforward neural network algorithm proposed by Huang et al. It has the characteristics of fast learning, good generalization performance, and a unique optimal solution compared with the traditional feedforward neural network algorithm [25]. To run ELM, the number of hidden-layer neurons (HLNs) should be set and the appropriate excitation function selected, while the bias of the hidden layer and the connection weight between the input and hidden layers are generated randomly during the operation of the algorithm. Without adjustment, the connection weight between the hidden and output layers can be obtained, thereby obtaining a unique global optimal solution.

In practice, there is no fixed theory for determining the number of HLNs. Generally, the stepwise trial method is adopted, which initially sets the range of the number of HLNs, then calculates the effect of the ELM model under each HLN within the range to select the optimal number of HLNs.

#### 2.4.2. Characteristic Wavelength Selection

If 200 wavelengths within a spectral range of 975–1645 nm are all adopted for full-spectra data modeling, the modeling speed will not only be affected by a large amount of data, but the effect of the calibration model will also be affected by the high correlation and the large amount of redundant information between different wavelengths, as well as the large amount of noise which will interfere with the establishment of the calibration model. Therefore, the wavelengths with minimum collinearity, the least redundancy, and the main effective information were selected from the original spectral data, and these few wavelengths were used to replace the original full-spectra data to establish the calibration model [26]. It was expected that the established model would be more robust without reducing the prediction ability of the model. In this work, uninformative variable elimination (UVE) and the successive projections algorithm (SPA) were used to select the characteristic wavelengths.

In the full spectra, there are some wavelengths that contain little or no valid information on the calibration model, and UVE can eliminate these wavelengths from the full spectra to improve model performance [27]. UVE is a wavelength selection algorithm based on the regression coefficient in the PLS model [28]. In the PLS model, the relationship between reference values *Y* and spectral matrix *X* (n × s) is Y=Xk+ε, where *k* is the regression coefficient and ε is the error.

The UVE algorithm is as follows: (i) A noise matrix *N* (n × s) with the same size as the spectral matrix *X* (n × s) is randomly generated, then *N* is combined with *X* to obtain *XN* (n × 2s). (ii) The PLS model is established by using the leave-one-out cross validation method based on *XN* and *Y*, and the corresponding regression coefficient matrix *k* (n × 2s) is obtained. (iii) The mean value *M* (1 × 2s) and the standard deviation *SD* (1 × 2s) of *k* (n × 2s) is calculated by column, and the *E*(*j*) of each column is calculated using Equation (2):

(2) $$E\left(j\right)=\frac{M\left(j\right)}{S\left(j\right)},\;j=1,2,\ldots ,2s-1,2s.$$

(iv) The maximum absolute value of *E*(*j*) in the interval [s + 1, 2s] is denoted as *E_max_*. (v) If $E\left(j\right)<\alpha E_{max}$ in the interval [1, s], then the *j*th wavelength in the spectral matrix is considered uninformative and eliminated from the dataset, and the rest of the wavelengths are chosen as characteristic wavelengths. In this work, α was set to 0.99.

The SPA is a forward feature variable selection algorithm [29]. The SPA compares the value of the projection vector by projecting the wavelength onto other wavelengths, and it takes the wavelength with the maximum projection vector value as the set of wavelengths to be selected. Finally, based on the calibration model, using root-mean-square error of leave-one-out cross validation (RMSECV) as the evaluation indicator, the characteristic wavelengths are selected from the set of selected wavelengths using the multiple linear regression method.

SPA selects the combination of variables with the least redundant information and minimum collinearity, and the number of characteristic wavelengths selected by SPA cannot be large.

#### 2.4.3. Model Assessment and Software

Model performance was assessed using the correlation coefficient of calibration (r_c_) and prediction (r_p_), the root-mean-square error of calibration (RMSEC) and prediction (RMSEP), and the residual prediction deviation (RPD) of the calibration and prediction models. Generally speaking, the larger the r_c_, r_p_, and RPD, the smaller the RMSEC and RMSEP, indicating the better performance of the model [30]. Especially when the RPD value is between 2.0 and 2.5, it indicates that the model works well and can be used for quantitative analysis. When the RPD value exceeds 2.5, it means that the model performs excellently [31].

Data processing of the hyperspectral images was performed in ENVI 5.3 (ITT, Visual Information Solutions, Boulder, CO, USA) and MATLAB R2010b (The MathWorks, Natick, MA, USA). The PLS model was built in Unscrambler 9.7 (CAMO Inc., Oslo, Norway). The ELM model was built in MATLAB R2010b. SPA, UVE, and visualization programs were all executed on MATLAB R2010b.

### 2.5. Image Processing

All pixels in the hyperspectral image data have a spectral reflectance curve with the full spectra [32]. The visualization of TN content distribution in soil by means of HSI technology has two important points. First, a fast and robust prediction model of TN content was established. Second, the spectral data of all pixels in the hyperspectral images were successively substituted into the established model to predict the content and obtain the grayscale image. Through the pseudocolor processing of the grayscale image, the visualization distribution map of TN content in soil was obtained.

## 3. Results and Discussion 

### 3.1. Models with Full Spectra

Firstly, the PLS and the ELM models were established with spectral data in the full spectra of each sample in the calibration set, then the two models were each used to predict the TN content of all samples in the prediction set. The performances of the two models are shown in Table 2.

The results indicate that both the PLS and ELM models have strong robustness in predicting TN content in soil, since their r_c_ and r_p_ were both over 0.9 and the RPD > 2.5. Meanwhile, the performance of the ELM model was superior to the PLS model, both in the calibration set and the prediction set. This may be because the ELM model considered the nonlinear information in the spectral data and used this information to obtain better predictive performance. Other scholars also found similar results. Shao et al. explored the nonlinear model of least-squares SVM (LS-SVM) for the measurement of soil N, P, and K on NIR spectroscopy and mid-infrared spectroscopy and found that, compared with the PLS model, the LS-SVM model provided a slight improvement of the prediction accuracy [33]. Cai et al. compared the prediction ability of different nonlinear models for measuring soil moisture content on visible and NIR (vis–NIR) spectroscopy, and the results showed that ELM performed better than the back-propagation neural network (BPNN), SVM, and RF [34]. This reflected that the ELM model has strong predictive and analytical capabilities for nonlinear problems. 

### 3.2. Characteristic Wavelength Selection

**Figure 3:**
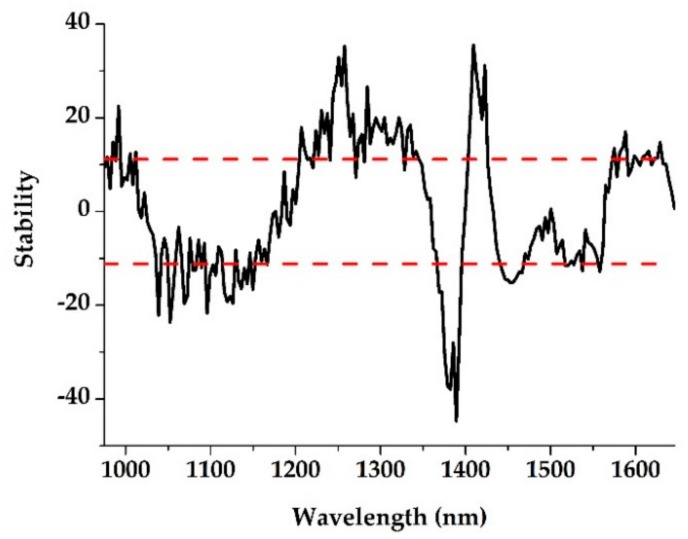
The stability distribution of TN using uninformative variable elimination (UVE).

In order to obtain a simpler and more reliable calibration model via reducing the number of wavelengths, UVE and SPA were applied to select the characteristic wavelengths with minimum collinearity and the main effective information from the full spectra. Figure 3 shows the stability values of soil TN in the wavelength range of 975–1645 nm using the UVE method.

The two dotted lines indicate the lower and upper cutoff values. The wavelengths corresponding to the stability variables outside the dotted line were selected as the characteristic wavelengths to establish the calibration model [35].

**Figure 4:**
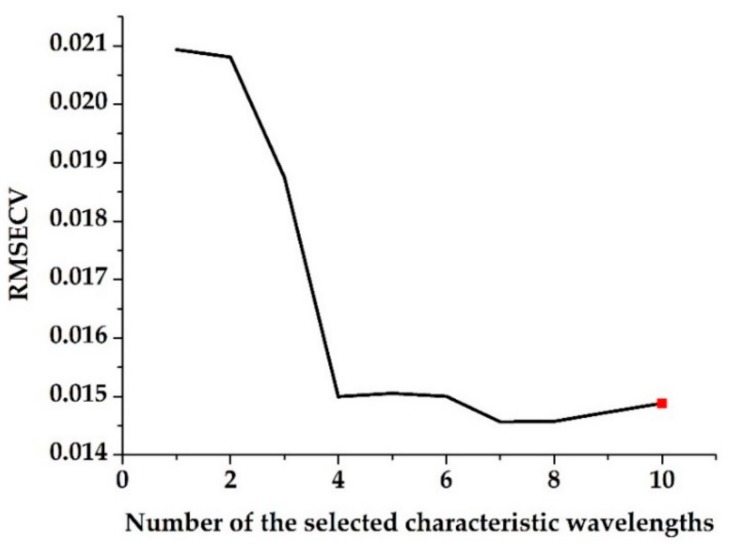
Root-mean-square error of leave-one-out cross validation (RMSECV) changes with the different numbers of selected characteristic wavelengths found using the successive projections algorithm (SPA).

Figure 4 shows the change in RMSECV with different numbers of selected characteristic wavelengths using the SPA method. RMSECVmin was set as the minimum value in the RMSECV sequence. The minimum number of characteristic wavelengths whose RMSECV values were not significantly greater than RMSECVmin was found using the F test at the significance level of α = 0.1 [36], thereby determining the number of selected characteristic wavelengths as 10.

**Figure 5:**
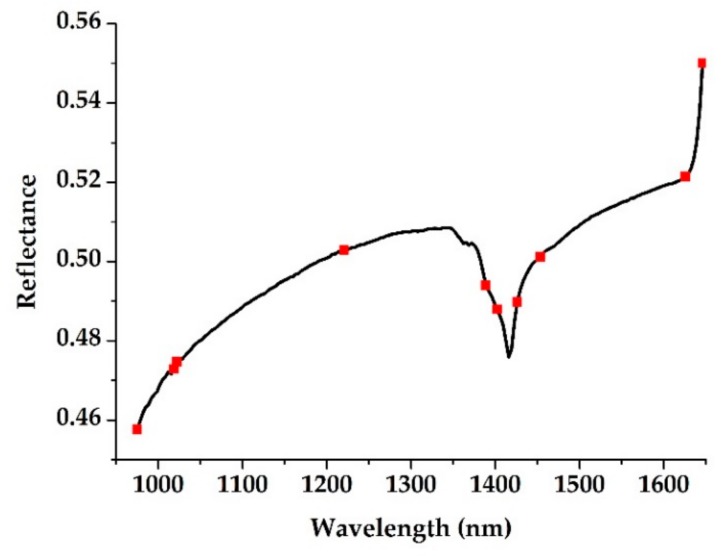
The characteristic wavelengths (square markers) selected using SPA.

Figure 5 shows the position of the selected characteristic wavelengths in the original spectrum.

### 3.3. Models with Characteristic Wavelengths

Based on the characteristic wavelengths selected by UVE and SPA, the models of UVE–PLS, SPA–PLS, UVE–ELM, and SPA–ELM were separately established. The performance of each model is shown in Table 3.

Firstly, the results showed that the UVE–PLS, SPA–PLS, UVE–ELM, and SPA–ELM models all had good prediction performances; the r_c_ and r_p_ values were all larger than 0.9; and the RPD values were all over 2.5. These results indicate that UVE or SPA can be used to select characteristic wavelengths instead of the full spectra in establishing the calibration and prediction models. Similar results were found by other scholars. Shen et al. used the PLS model combined with UVE and SPA to estimate the soil carbon content on NIR spectroscopy, and the results showed that UVE and SPA were beneficial and necessary for soil carbon modeling on NIR spectroscopy [37]. Yang et al. used UVE and SPA to extract characteristic wavelengths from vis–NIR spectral data, and these characteristic wavelengths were used to model and predict total nitrogen, total carbon, organic carbon, and inorganic carbon in soil. The prediction results showed that UVE can reduce wavelength variables significantly while retaining good model prediction accuracy [27].

Secondly, under the same characteristic wavelength selection method, the performances of the ELM models were better than the PLS models, which was similar to the conclusion obtained in Section 3.1 “Models with Full Spectra”.

Thirdly, the performances of the models based on full spectra (refer to Table 2) and models based on the characteristic wavelengths selected by UVE were better than those based on the characteristic wavelengths selected by SPA. This may be because the characteristic wavelengths selected by the SPA based on the calibration model were unable to represent the new variables in the prediction model effectively, as the number of wavelengths was greatly reduced. As mentioned above, Shen et al. and Yang et al. found similar results when measuring the soil carbon content and the soil TN, respectively [27,37]. The results showed that the characteristic wavelength selection model based on UVE was more accurate than the model based on SPA in terms of the higher RPD. This can be explained by the fact that the SPA method tended to select unstable variables and to remove some important relevant variables when operating on the full spectra [27,37].

**Figure 6:**
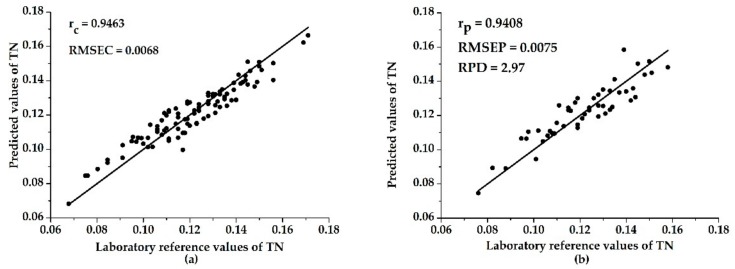
The scatter plots of the calibration set (**a**) and the prediction set (**b**) in the UVE–ELM model.

In the end, the performances of models based on the characteristic wavelengths selected using UVE were more accurate and robust than those based on full spectra, indicating that UVE is an efficient method for characteristic wavelength selection. The UVE–ELM model performed best out of all the models, with an r_c_ of 0.9463, r_p_ of 0.9408, RMSEC of 0.0068, RMSEP of 0.0075, and RPD of 2.97 (shown in Figure 6). In comparison, Yang et al. adopted the UVE–SPA–LS-SVM model to estimate the soil TN on vis–NIR spectroscopy, and the performance of the prediction model was r_p_ = 0.8913 and RPD = 2.009 [38]. Yuan et al. used the PLS model combined with the spectral pretreatment method to predict the content of TN in soil on NIR spectroscopy, with an r_p_ of 0.9454 [39]. This showed that the performances of different prediction models on different spectral ranges were almost similar. In summary, the characteristic wavelength selection methods were effective when using HSI technology to detect the TN content in soil, and the UVE method was more suitable for this work.

### 3.4. Image Visualization 

**Figure 7:**
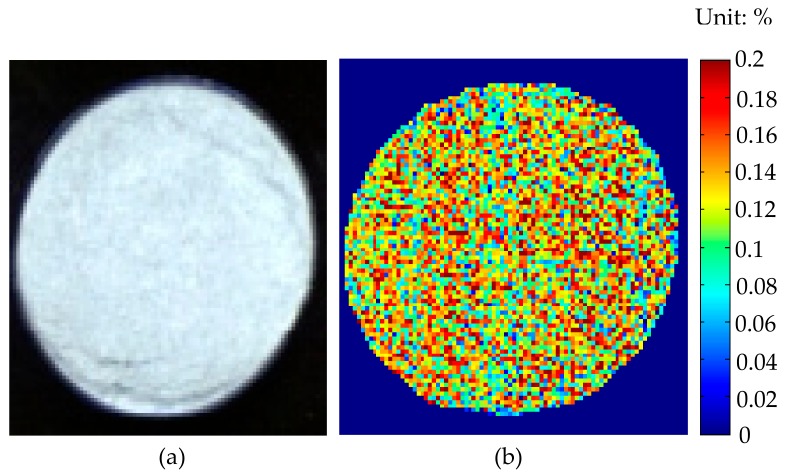
(**a**) The hyperspectral image and (**b**) the corresponding TN content distribution map visualized based on the UVE–ELM model.

The realization of hyperspectral image visualization was first based on the calibration model established according to the average spectrum of the sample pixels and the corresponding reference values. Then, the model was applied to predict the reference value of each pixel. As mentioned above, the UVE–ELM model performed best among all the models, so the UVE–ELM model was used to estimate the TN value at each pixel of the soil hyperspectral image. A soil sample was randomly selected from the prediction set, and the hyperspectral image of the sample was firstly masked to make the reflectance of the background and the unwanted part in the image become 0. Then, the spectral data of each pixel in the hyperspectral image was substituted into the UVE-ELM model to predict the TN value at each pixel, and the grayscale image was obtained. Finally, the color of all pixels was varied between red and blue through an image processing program, indicating the different TN contents from high to low, respectively. The hyperspectral image of the soil sample and the corresponding TN content distribution map are shown in Figure 7.

According to the data analysis in the image processing program, the predicted TN values of most pixels (>85%) were within the TN reference value of the calibration set (0.0678%–0.1710%), and the corresponding colors displayed on the distribution map were mostly between cyan and light red. The average predicted TN value of all pixels was 0.119%, while the reference value of TN content in the soil sample was 0.108%. Since the reference value of TN content in each pixel was unknown, it was difficult to verify the accuracy of the predicted value of each pixel. In practical applications, the reference value range of the calibration set should be ensured to be as large as possible and the reference value range of the prediction set should be included, so that the accuracy of prediction can be improved. Hyperspectral image visualization provided an intuitive representation of the TN content distribution map in soil, so as to make corresponding decisions.

## 4. Conclusions

The results indicate that the combination of HSI technology and chemometrics was an effective method to measure TN content in soil. Compared with the full-spectra model, UVE improved the performance of PLS and ELM, which indicates that UVE is an efficient method for characteristic wavelength selection. It also showed that, compared with full-spectra modeling, modeling with a limited number of characteristic wavelengths can achieve a better prediction effect. This provides a theoretical and methodological basis for instrument development based on the selection of characteristic wavelengths, thereby reducing production costs. The visualization distribution map of TN content in soil, which was obtained based on the UVE–ELM model, provides a foundation for future hyperspectral remote sensing identification and inversion of TN content in soil. This is helpful for monitoring the change of TN content in soil in real time. It is of great significance for the future use of an online monitoring system for soil nutrient content at a field scale.

## Figures and Tables

**Table 1:** Reference values of total nitrogen (TN) content in the 150 soil samples.

Sample Set	Number	Range (%)	Mean (%)	SD ^1^ (%)
Calibration set	100	0.0678–0.1710	0.1216	0.0201
Prediction set	50	0.0760–0.1580	0.1197	0.0223

^1^ SD = Standard deviation.

**Table 2:** The performances of the partial least squares (PLS) model and the extreme learning machine (ELM) model with full spectra.

Model	LVs ^1^/HLNs ^2^	Calibration		Prediction		
		r_c_	RMSEC (%)	r_p_	RMSEP (%)	RPD
PLS	6	0.9276	0.0077	0.9218	0.0086	2.59
ELM	24	0.9383	0.0072	0.9347	0.0079	2.82

^1^ LVs is the number of latent variables in the PLS model. ^2^ HLNs is the number of hidden-layer neurons in the ELM model.

**Table 3:** The performance of the PLS and ELM models with the characteristic wavelengths selected using UVE and SPA.

Model	Calibration		Prediction		
	r_c_	RMSEC (%)	r_p_	RMSEP (%)	RPD
UVE–PLS	0.9293	0.0074	0.9266	0.0083	2.69
SPA–PLS	0.9310	0.0076	0.9150	0.0089	2.51
UVE–ELM	0.9463	0.0068	0.9408	0.0075	2.97
SPA–ELM	0.9346	0.0074	0.9196	0.0087	2.56
